# The effect of CT texture-based analysis using machine learning approaches on radiologists' performance in differentiating focal-type autoimmune pancreatitis and pancreatic duct carcinoma

**DOI:** 10.1007/s11604-022-01298-7

**Published:** 2022-06-21

**Authors:** Kenta Anai, Yoshiko Hayashida, Issei Ueda, Eri Hozuki, Yuuta Yoshimatsu, Jun Tsukamoto, Toshihiko Hamamura, Norihiro Onari, Takatoshi Aoki, Yukunori Korogi

**Affiliations:** 1 Department of Radiology, University of Occupational and Environmental Health, 1-1, Iseigaoka, Yahatanishi-ku, Kitakyushu, Fukuoka 807-8555 Japan; 2 Department of Radiology, Kyushu Rosai Hospital, Moji Medical Center, 3-1, Higashiminatomachi, Moji-ku, Kitakyushu, Fukuoka 801-8502 Japan

**Keywords:** Machine learning, Autoimmune pancreatitis, Pancreatic duct carcinoma, Texture analysis, CT

## Abstract

**Purpose:**

To develop a support vector machine (SVM) classifier using CT texture-based analysis in differentiating focal-type autoimmune pancreatitis (AIP) and pancreatic duct carcinoma (PD), and to assess the radiologists’ diagnostic performance with or without SVM.

**Materials and methods:**

This retrospective study included 50 patients (20 patients with focal-type AIP and 30 patients with PD) who underwent dynamic contrast-enhanced CT. Sixty-two CT texture-based features were extracted from 2D images of the arterial and portal phase CTs. We conducted data compression and feature selections using principal component analysis (PCA) and produced the SVM classifier. Four readers participated in this observer performance study and the statistical significance of differences with and without the SVM was assessed by receiver operating characteristic (ROC) analysis.

**Results:**

The SVM performance indicated a high performance in differentiating focal-type AIP and PD (AUC = 0.920). The AUC for all 4 readers increased significantly from 0.827 to 0.911 when using the SVM outputs (*p* = 0.010). The AUC for inexperienced readers increased significantly from 0.781 to 0.905 when using the SVM outputs (*p* = 0.310). The AUC for experienced readers increased from 0.875 to 0.912 when using the SVM outputs, however, there was no significant difference (*p* = 0.018).

**Conclusion:**

The use of SVM classifier using CT texture-based features improved the diagnostic performance for differentiating focal-type AIP and PD on CT.

## Introduction

Autoimmune pancreatitis (AIP) is a rare chronic pancreatitis marked by pancreatic enlargement, irregular pancreatic duct stenosis, and elevated serum immunoglobulin 4 (IgG4) levels, mediated by autoimmune mechanisms [1]. AIP doesn't have any characteristic clinical manifestations and is often misdiagnosed as pancreatic duct carcinoma (PD), and therefore, some patients undergo unnecessary surgery as a result [2]. On the other hand, the imaging findings associated with serological examinations (IgG4 and Ca19-9) plays an important role in the distinction between these entities. Since, 7–10% of pancreatic cancer patients show high serum IgG4 levels, and some AIP patients may exhibit equivocal serum IgG4 levels and elevated levels of CA19-9, the utility of the serological examinations may be limited [3–6]. Therefore, the diagnostic imaging is the key in differentiating between AIP and PD.

AIP is categorized into Type 1 and Type 2. Type 1 is common, and is regarded as a prototype of IgG4-related disease, with high serum levels of IgG4 (> 140 mg/dl), IgG4-positive plasma cell infiltration, and sclerosis. Type 2 is considered granulocytic epithelial lesions. Both types can present various morphological changes of the pancreas, which include diffuse, focal/mass-forming, or multifocal disease [7–9]. Focal-type AIP is accounting for 33–41% of the cases of AIP [10, 11]. Differentiation between focal-type AIP and PD by conventional imaging methods can be difficult.

Multiple imaging techniques, including CT, MRI, and 18F-fluorodeoxyglucose positron-emission tomography/computerized tomography (18F-FDG PET/CT), have been used for solving this problem [12–14].

Recent advances and application developments of radiomics have helped the improvement of disease prediction and classification accuracy in differentiating tumors. Many researchers have used texture-based analysis in an attempt to differentiate tumors using machine learning [15–17]. However, to our knowledge, only a few studies have investigated CT texture-based analysis to differentiate focal-type AIP and PD via a machine learning approach [17–19]. In addition, the impact of machine learning model on observer performance has not been reported in previous studies.

The purpose of this study was to develop a CT texture-based support vector machine (SVM) classifier in differentiating focal-type AIP and PD via a machine learning approach, and to compare the diagnostic performance with and without the SVM classifier.

## Materials and methods

### Subjects

Our institutional review board approved this retrospective study and informed consent from patients was waived. We retrospectively reviewed the medical records of all patients who had undergone abdominal dynamic contrast-enhanced CT at our hospital between March 2005 and August 2019, and selected for this analysis the patients who met the following criteria: (a) fulfilled with the International Consensus Diagnostic Criteria (ICDC) for AIP [20] or Revised Japanese Pancreas Society criteria of AIP [21]; or (b) histopathologically diagnosed with PD after surgical resection at our institution. Finally, 20 patients with focal-type AIP (11 men, 9 women; mean age, 65.3 years; range, 46–81 years) and 30 patients with PD (18 men, 12 women; mean age, 66.9 years; range, 52–82 years) were included for this study. In the AIP group, 14 patients were diagnosed on the basis of ICDC, all of whom had type 1 AIP (definite, 12; probable, 2). All patients were diagnosed on the basis of the Japanese diagnostic criteria (definite, 12; probable, 4; possible, 4). In the PD group, the number of patients with clinical T stages 1a, 1b, 1c, 2, 3 and 4 were 0, 0, 8, 22, 0 and 0, respectively. All patients with focal-type AIP and PD underwent CT examinations before receiving therapy. Intergroup comparisons between focal-type AIP and PD were performed using Chi-square test for categorical variables and the Student’s t test for numeric variables.

### Image acquisition

In the AIP group, the number of patients scanned on a 16-slice MDCT scanner (Aquilion^®^, Toshiba Medical Systems, Tokyo, Japan), a 32-slice MDCT scanner (Aquilion^®^, Toshiba Medical Systems, Tokyo, Japan), a 64-slice MDCT scanner (Aquilion^®^, Toshiba Medical Systems, Tokyo, Japan), and a 320-slice MDCT scanner (Aquilion ONE^®^, Toshiba Medical Systems, Tokyo, Japan) were 4, 1, 12, and 3, respectively. In the PD group, the number of patients scanned on the 64-slice MDCT scanner (Aquilion^®^, Toshiba Medical Systems, Tokyo, Japan) and the 320-slice MDCT scanner (Aquilion ONE^®^, Toshiba Medical Systems, Tokyo, Japan) were 21 and 9, respectively.

CT data of the 16-slice MDCT scanner were acquired using the following parameters: tube voltage, 120 kV; tube current, 200 mA without automatic exposure control; gantry rotation speed, 0.5 s; collimation, 16 × 2 mm; and beam pitch, 0.938. CT data of the 32-slice MDCT scanner were acquired using the following parameters: tube voltage, 120 kV; tube current, 270 mA without automatic exposure control; gantry rotation speed, 0.5 s; collimation, 32 × 1 mm; and beam pitch, 0.828. CT data of the 64-slice MDCT scanner were acquired using the following parameters: tube voltage, 120 kV; tube current, automatic exposure control with a fixed noise index (SD 10 at 3 mm thickness) gantry rotation speed, 0.5 s; collimation, 32 × 1 mm; and beam pitch, 0.828. CT data of the 320-slice MDCT scanner were acquired using the following parameters: tube voltage, 120 kV; tube current, automatic exposure control with a fixed noise index (SD 10 at 3 mm thickness); gantry rotation speed, 0.5 s; collimation, 80 × 0.5 mm; and beam pitch, 0.813.

After unenhanced images had been acquired, non-ionic contrast material (550 mg I/kg body weight) was injected through the peripheral venous line within 30 s. Arterial phase imaging was performed with fixed delay or bolus triggering, usually between 35 and 40 s post-injection, and portal phase imaging was performed at 60–70 s.

The helical data of the 16-slice MDCT scanner were reconstructed using FBP with our standard reconstruction kernel (FC10). The helical data of the 32- and 64-slice MDCT scanner were reconstructed using FBP with our standard reconstruction kernel (FC14). The helical data of the 320-slice MDCT scanner were reconstructed using AIDR3D (weak or mild setting) with our standard reconstruction kernel (FC14). The image reconstruction was performed in a 32 to 45 cm display field of view depending on the patient's physique. CT image analysis was performed using arterial and portal phase images with a 3 mm section thickness at 3-mm intervals.

### Texture feature extraction

**Fig. 1:**
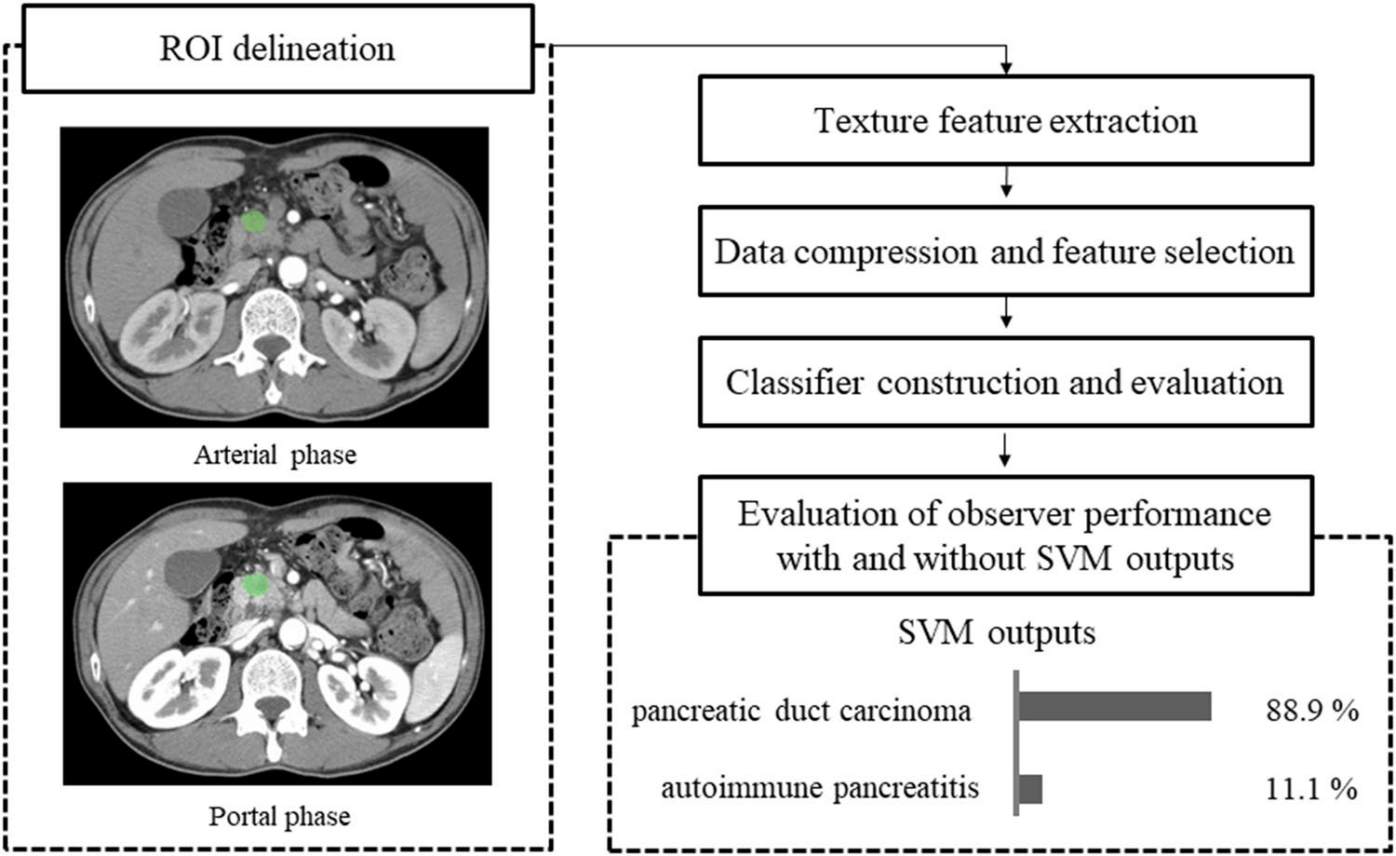
Overall flowchart explaining the process in this study. First, texture features were extracted from arterial and portal phase CT images using two-dimensional analysis. Then, we conducted data compression and feature selections using principal component (PC) analysis. Subsequently, the support vector machine (SVM) classifier was conducted and the diagnostic accuracy was evaluated. Finally, the effect of SVM on observers’ performance with SVM outputs was also evaluated

**Fig. 2:**
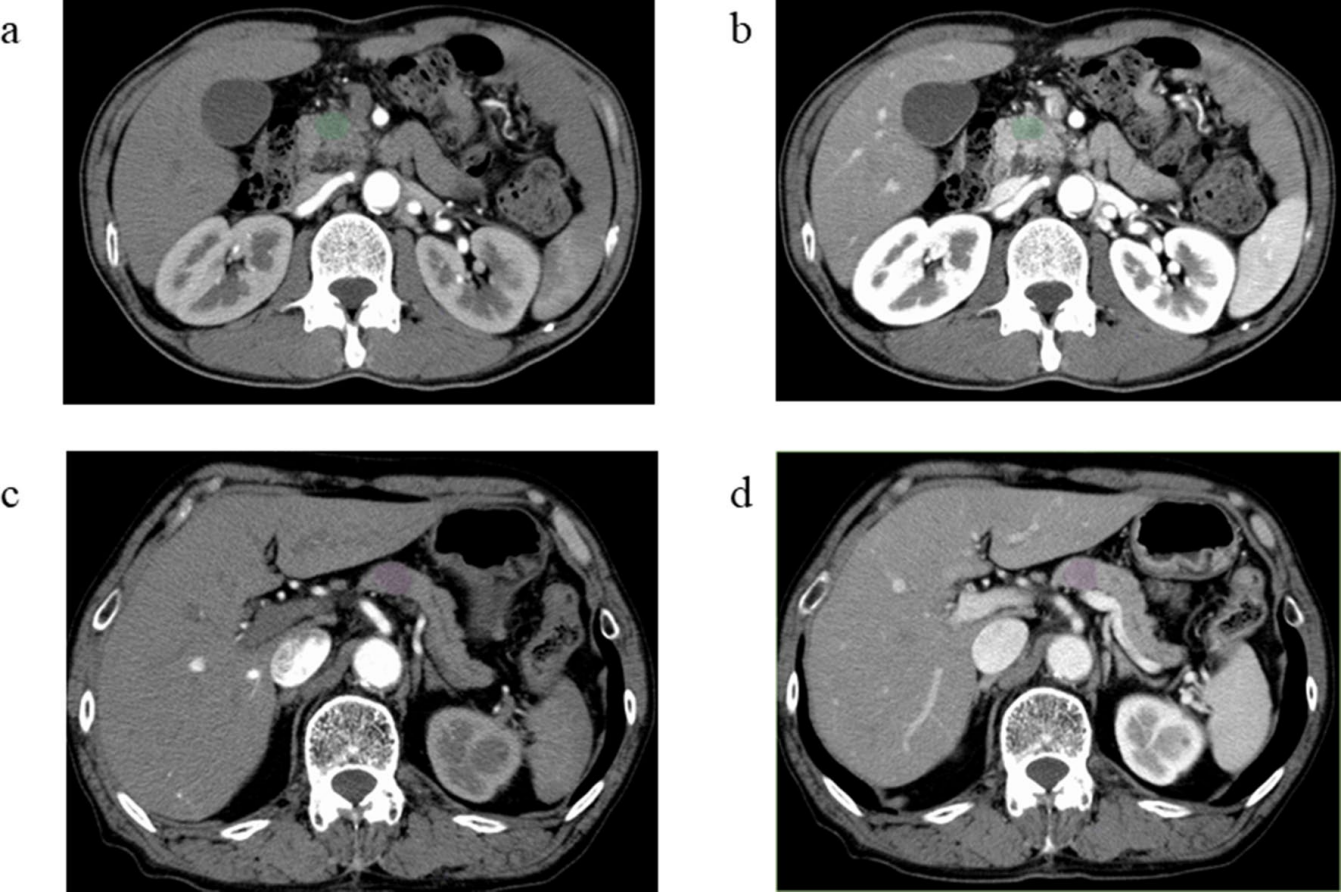
Arterial phase (**a**) and portal phase (**b**) images of a patient with pancreatic duct carcinoma (PD). Arterial phase (**c**) and portal phase (**d**) images of a patient with focal-type autoimmune pancreatitis (AIP). A manually defined ROI is drawn in the pancreatic lesion by LIFEx software

The overall flowchart explaining the process in this study is shown in Fig. 1. We chose an axial image slice of arterial and portal phase on the basis of the maximum diameter of the lesion. Texture parameter calculation was performed with the software LIFEx ([20]; version 4.00, available at https://www.lifexsoft.org/). Two-dimensional segmentation was performed by manually drawing a region of interest (ROI) around the lesion outline as big as possible by one radiologist (K.A) (Fig. 2), and verified and confirmed by another radiologist (T.H); both were blinded to the patients’ clinical outcomes. First, we calculated 37 texture features defined in LIFEx version 4.00 software. The texture features of each ROI were then obtained, we excluded the data of six texture features (i.e., GLRLM_LGRE, GLRLM_LRLGE, GLRLM_SRLGE, GLZLM_LGZE, GLZLM_LZLGE and GLZLM_SZLGE) since they showed zero values in all of the ROIs from pancreatic lesions. Therefore, we used the 31 textural feature data set for this study. The 31 texture features used in this study are shown in Table 1. Finally, we used a total of 62 texture features from arterial and portal phases.

**Table 1:** Summary of the texture features for the analysis

Feature name	Symbol/abbreviation
Geometry based and histogram based features	
Skewness	–
Kurtosis	–
Entropy log10	–
Entropy log2	–
Energy	–
Gray-level co-occurrence matrix (GLCM)	
Homogeneity	–
Energy	–
Contrast	–
Correlation	–
Entropy log10	–
Entropy log2	–
Dissimilarity	–
Neighborhood gray-level different matrix (NGLDM)	
Contrast	–
Coarseness	–
Busyness	–
Grey level run length matrix GLRLM)	–
Short-Run Emphasis	SRE
Long-Run Emphasis	LRE
Low Gray-level Run Emphasis	LGRE
High Gray-level Run Emphasis	HGRE
Short-Run High Gray-level Emphasis	SRHGE
Long-Run Low Gray-level Emphasis	LRLGE
Long-Run High Gray-level Emphasis	LRHGE
Gray-Level Non-Uniformity for run	GLNU
Run Length Non-Uniformity	RLNU
Run Percentage	RP
Grey level zone length matrix (GLZLM)	
Short-Zone Emphasis	SZE
Long-Zone Emphasis	LZE
High Gray-level Zone Emphasis	HGZE
Short-Zone High Gray-level Emphasis	SZHGE
Long-Zone Low Gray-level Emphasis	LZLGE
Long-Zone High Gray-level Emphasis	LZHGE
Gray-Level Non-Uniformity for zone	GLNU
Zone Length Non-Uniformity	ZLNU
Zone Length Non-Uniformity Zone Percentage	ZP

### Data compression and feature selection

To keep the interpretability as well as the high classification performance of the classifier, we conducted data compression and feature selections by using principal component analysis (PCA). Since we could not make any prior selection of features based on their mathematical definitions or previous studies, we compressed all of the 62 features by extracting original features as well as new features with PCA. After compressing the data, we selected the principal components (PC) which were able to explain at least 80% of the total variance and plotted their scores. We then selected the best two components with scores that clearly separated between the AIP group and PD group subjectively. We also performed Wilcoxon–Mann–Whitney tests to assess objectively whether the score differences were significant between the two groups. *p* < 0.025 was considered statistically significant.

### SVM classifier construction and evaluation

We constructed an SVM classifier to predict the disease type (AIP or PD) using the two principal components selected in the previous section. SVM is a discriminant function that uses multiple features to classify into two classes. When the e1071 package (https://cran.r-project.org/web/packages/e1071/index.html) is used, the SVM model function outputs a decision value for each region. Each region is classified according to whether the output value of the discriminant function exceeds 0 or not. When the output value is greater than zero, unknown data is classified as AIP. When the output value is less than zero, unknown data is classified as PD. In the training phase, we constructed many classifiers with various hyper parameter combinations. Then we chose the best model that was able to classify the regions with the smallest classification error (i.e. the highest accuracy). The tunable hyper-parameters in this work were the cost of misclassification (*C*) and the inverse of the standard deviation of the RBF kernel (*γ*). Coarse grid searches were used to tune these parameters and threefold cross-validation was conducted to find the average performance of the classifier. Sensitivity, specificity, accuracy, positive predictive value (PPV) and negative predictive value (NPV) were calculated using this classification result. On the other hand, receiver operating characteristic (ROC) analysis was performed using the output data of the discriminant function, and the area under the curve (AUC) was calculated. For observer performance study, a model was trained using the 2 folds as training data. The resulting model was validated on the remaining part of the data (i.e. a test data) and extracted output. The data analyses were conducted by using the R software program (version 3.4.1; www.R-project.org).

### Observer performance study

We used an independent test method for diagnostic performance evaluation, and one radiologist lined up 20 patients with focal-type AIP and 30 patients with PD in random order. Four radiologists with 5, 6, 24 and 30 years of experience participated in the observer performance study. The performance of the SVM classifier and clinical data were not informed. Assessment of each radiologist’s performance was determined by receiver operating characteristic (ROC) analysis with a continuous rating scale with a 0–100 scale. First, the readers reviewed an axial image slice of the arterial and portal phase of the lesion without SVM outputs. Axial images were chosen for the pancreas masses avoiding other key findings (such as lymph node (LN) swellings, vascular invasion or pancreatic duct dilatations). Subsequently, SVM outputs (the percentage of probability for each category) were presented to the observers via the bar graph, and a second observer performance study with SVM outputs was performed. The time interval between the first and second review was more than one month. The performance of the 4 readers was evaluated in terms of sensitivity, specificity, accuracy, PPV and NPV, and the AUC of the ROC. We divided readers into two groups, experienced and inexperienced, and the statistical significance of the differences between AUC values for radiologists with and without SVM outputs was evaluated by DeLong's test. For the both classifier performance and the observer performance, the data analyses were conducted by using the R software program (version 3.4.1; www.R-project.org). The sensitivities, specificities, PPV, NPV, and AUC of the SVM classifier were calculated automatically by the R software.

## Results

The clinical characteristics of 20 focal-type AIP and 30 PD patients are summarized in Table 2. No significant differences were found in age, sex or lesion size between the two groups.

**Table 2:** Clinical data of patients with focal-type autoimmune pancreatitis (AIP) and pancreatic duct carcinoma (PD)

	Focal-type AIP *n *= 20 (± SD)	PD *n* = 30 (± SD)	*p*
Age (y)	65.25 ± 9.40	66.87 ± 8.45	0.539
Male/Female	11/9	18/12	0.953
Lesion size (mm)	33.54 ± 13.62	26.96 ± 7.78	0.061
IgG4 > 280 (mg/dl)	8		
280≧IgG4≧135 (mg/dl)	7		
Narrowing of main pancratic duct on ERP/MRCP	18		
Pathological findings	9		
Other organ involvement	6		
Effectiveness of steroid therapy	15		
*cT Stage*^a^			
T1a		0	
T1b		0	
T1c		8	
T2		22	
T3		0	
T4		0	

*ERP* endoscopic retrograde pancreatography, *MRCP* magnetic resonance cholangiopancreatography

^a^According to the 8th edition of the TNM classification of malignant tumors

**Fig. 3:**
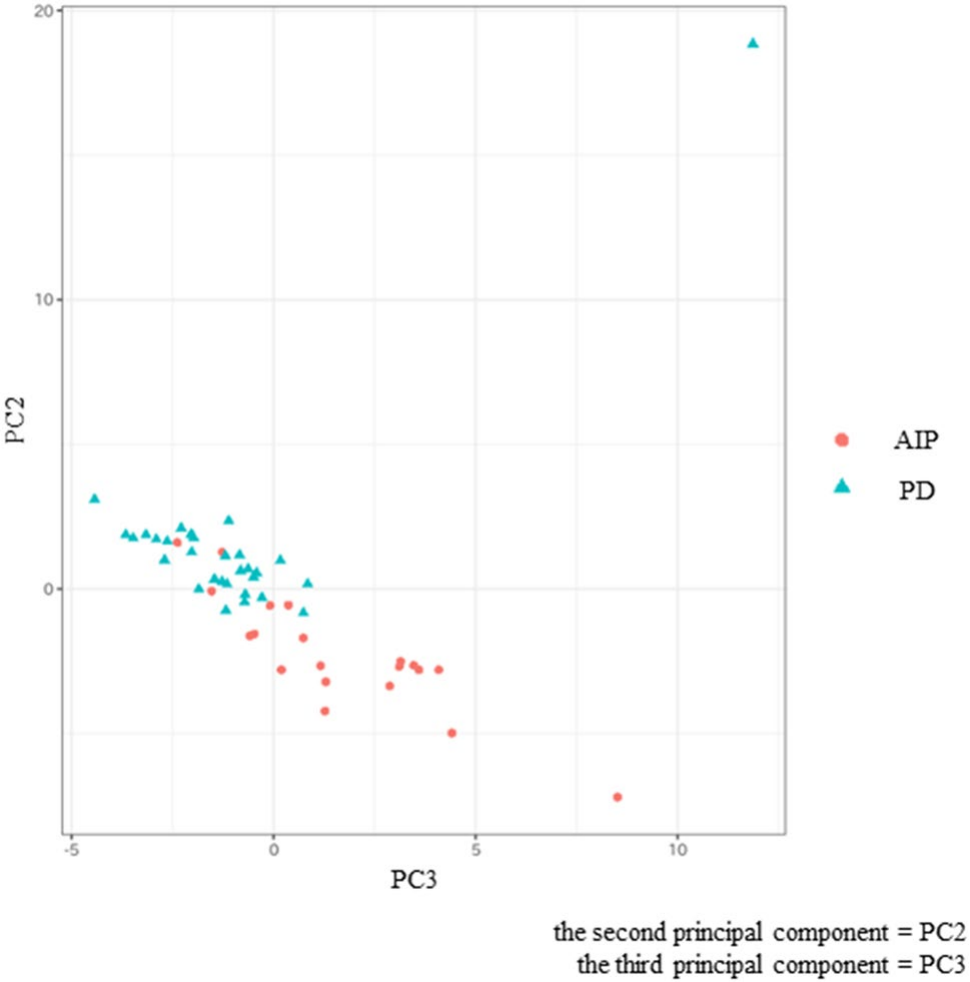
Scatterplot of the two principal components (PCs). Each dot represents an autoimmune pancreatitis (AIP) (red circle) or a pancreatic duct carcinoma (PD) (blue triangle). Dots were clearly separated between the AIP and the PD groups

**Fig. 4:**
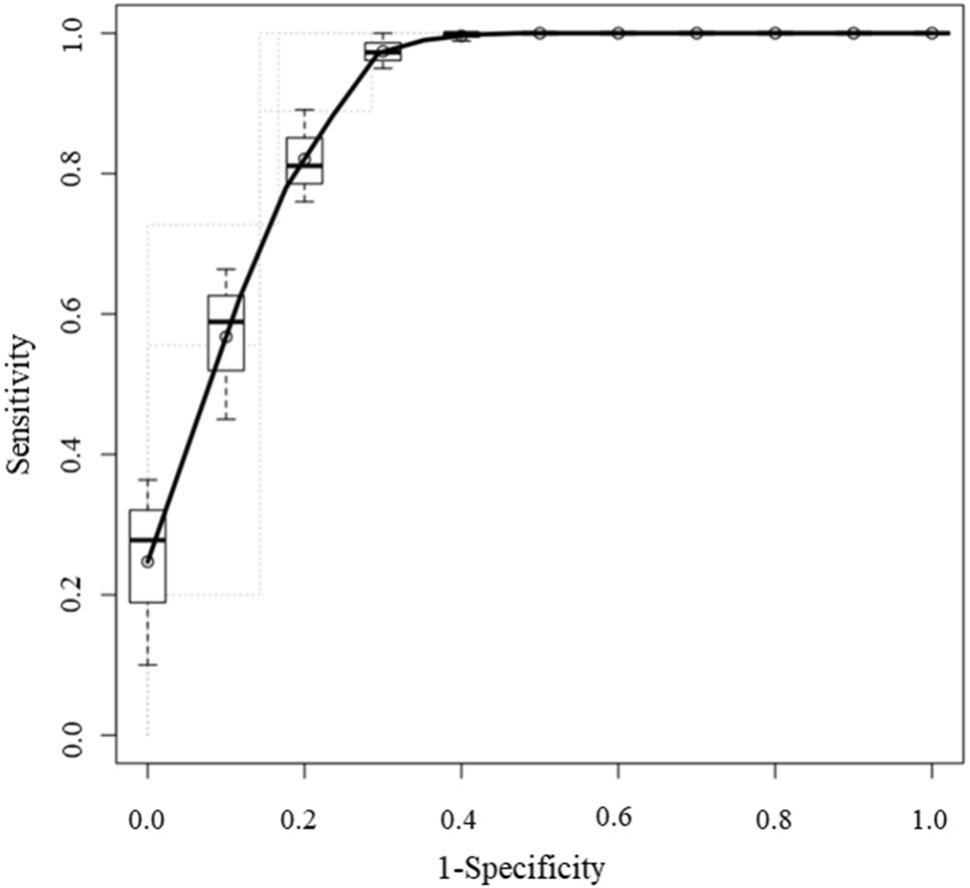
The receiver operating characteristic curve (ROC) for differentiating focal-type autoimmune pancreatitis (AIP) and pancreatic duct carcinoma (PD) of the support vector machine (SVM) classifier. The areas under the curve (AUC) were 0.920

We chose the best combination of principal components that could separate AIP group and PD group by plotting their scores (Fig. 3). As showing on Fig. 3, the combination of the second principal component (PC2) and the third principal component (PC3) was chosen because they could clearly separate two groups subjectively. The scores of PC2 and PC3 showed significant differences in focal-type AIP and PD (both *p* < 0.001) (Table 3). The top three texture features in PC2 loadings were arterial phase Skewness, portal phase NGLDM Coarseness and portal phase Skewness. The bottom three texture features in PC2 loadings were arterial phase GLRLM HGRE, arterial phase GLZLM HGZE and arterial phase GLRLM SRHGE (Table 4). The top three texture features in PC3 loadings were portal phase GLRLM RLNU, portal phase GLZLM GLNU and portal phase GLRLM GLNU. The bottom three texture features in PC3 loadings were arterial phase NGLDM Coarseness, arterial phase NGLDM Contrast and arterial phase GLRLM SRE (Table 5). ROC curves were adopted to determine the diagnostic performance of SVM classifiers in differentiating focal-type AIP and PD (Fig. 4). The parameters of SVM classifier including AUC, sensitivity (%), specificity (%), accuracy (%), PPV (%), and NPV (%) were 0.920, 100.0, 75.0, 90.0, 85.7 and 100.0%, respectively.

**Table 3:** Comparison of selected principal component (PC) scores between focal-type autoimmune pancreatitis (AIP) and pancreatic duct carcinoma (PD)

	Focal-type AIP (*n* = 20)	PD (*n* = 30)	*p*
PC2 score	−2.26 ± 2.03	1.50 ± 3.42	< .001*
PC3 score	1.59 ± 2.58	−1.06 ± 2.75	< .001*

*A significant difference (*p* < 0.025)

**Table 4:** Loadings of the second principal component (PC2)

Texture feature	PC2 loading
ap Skewness	0.1995
pp NGLDM Coarseness	0.1721
pp Skewness	0.1617
ap Kurtosis	0.1182
pp NGLDM Contrast	0.0855
・・・	・・・
ap GLCM Entropy log2	−0.2055
ap GLCM Entropy log10	−0.2056
ap GLRLM SRHGE	−0.2157
ap GLZLM HGZE	−0.2356
ap GLRLM HGRE	−0.2377

*ap* arterial phase, *pp* portal phase

**Table 5:** Loadings of the third principal component (PC3)

Texture feature	PC3 loading
pp GLRLM RLNU	0.2366
pp GLZLM GLNU	0.2332
pp GLRLM GLNU	0.2296
ap GLRLM RLNU	0.2249
ap Kurtosis	0.2172
・・・	・・・
pp NGLDM Coarseness	−0.1814
ap GLZLM HGZE	−0.1819
ap GLRLM SRE	−0.1839
ap NGLDM Contrast	−0.1871
ap NGLDM Coarseness	−0.2567

*ap* arterial phase, *pp* portal phase

**Fig. 5:**
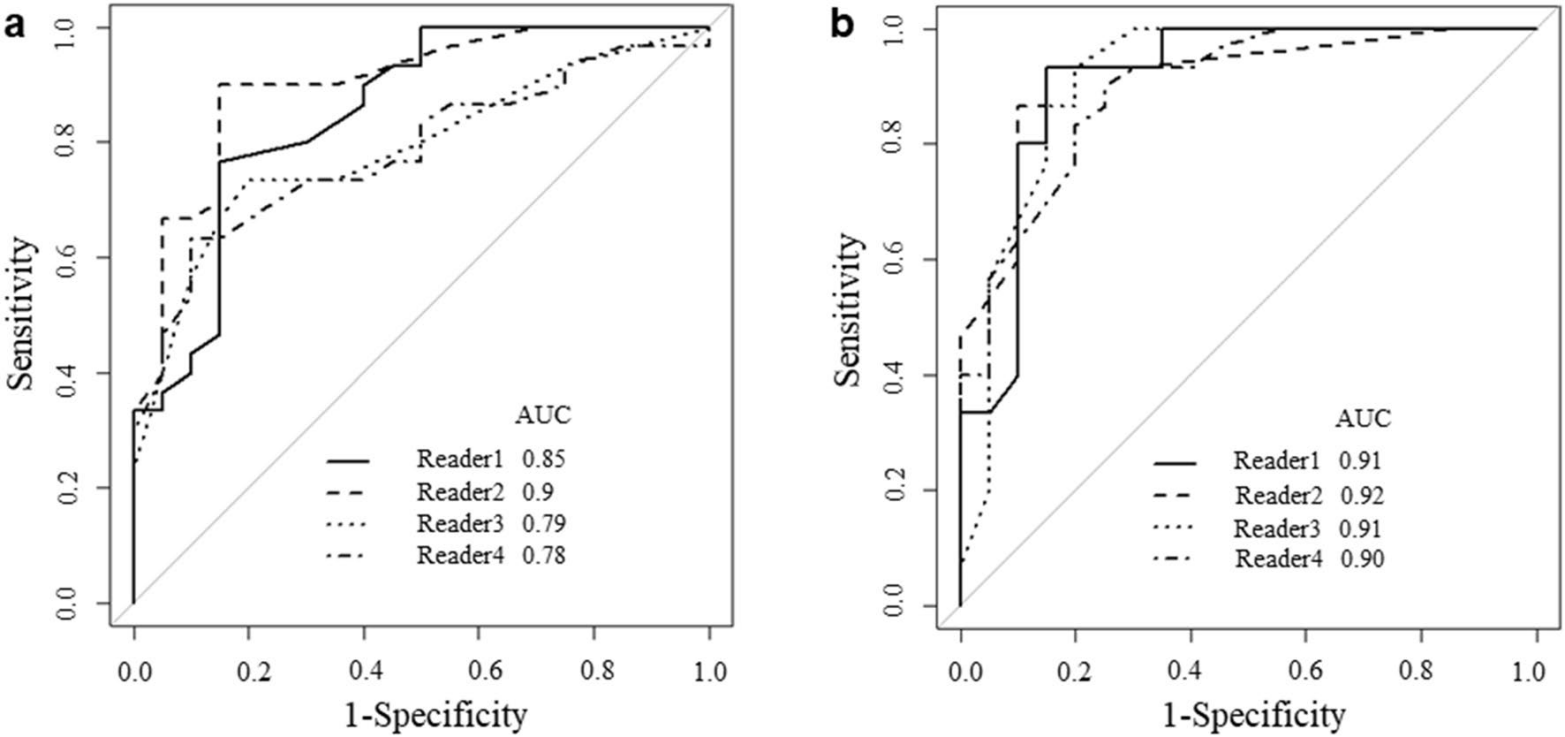
**a** Receiver operating characteristic curves (ROCs) for differentiating focal-type autoimmune pancreatitis (AIP) and pancreatic duct carcinoma (PD) of all the readers with support vector machine (SVM) outputs. The areas under the curves (AUCs) of the 4 readers were 0.85, 0.90, 0.79, and 0.78, respectively. **b** ROCs for differentiating focal-type AIP and PD of all the readers with SVM outputs. The AUCs of the 4 readers were 0.91, 0.92, 0.91, and 0.90, respectively

ROC curves were adopted to determine the diagnostic performance of all readers in differentiating focal-type AIP and PD (Fig. 5). The AUC without and with SVM outputs for each radiologist are shown in the Table 6. The AUC of all radiologists without SVM outputs were 0.827 and those with were 0.911 (*p* = 0.010). Divided into two groups, the AUC of the experienced group without SVM outputs was 0.875 and that with was 0.912 (*p* = 0.310). The AUC of the inexperienced group with SVM outputs was 0.781 and that with was 0.905 (*p* = 0.018).

**Table 6:** Observer performance for differentiating focal-type autoimmune pancreatitis (AIP) and pancreatic duct carcinoma (PD) with and without support vector machine (SVM) outputs

Observer	No.1	No.2	No.3	No.4	All	Experienced group (No.1 and 2)	Inexperienced group (No.3 and 4)
Exp. years	30	24	6	5			
AUC without SVM	0.852	0.900	0.789	0.783	0.827*	0.875	0.781*
AUC with SVM	0.912	0.918	0.913	0.898	0.911*	0.912	0.905*

*AUC* the area under ROC curve

*A significant difference (*p* < 0.05)

## Discussion

We developed an SVM classifier to differentiate focal-type AIP and PD and evaluated the diagnostic performance of readers with and without SVM outputs. To our knowledge, there is no prior study dealing with observer performance study for CT texture-based analysis in differentiating focal-type AIP and PD using machine learning approaches. Although the SVM outputs improved the diagnostic performance for all four readers including the experienced radiologists, an observer category with less experience benefited from the SVM outputs more. The AUC for inexperienced radiologists with the SVM outputs (0.905) was higher than that for experienced radiologists without the SVM outputs (0.875). These results may suggest that the SVM classifier may be more useful for inexperienced radiologists and the radiologist’s performance in differentiating focal-type AIP and PD depends on their experience.

In machine learning, SVMs are learning algorithms for analyzing data used for classification. The SVM can build a model that classifies new data into different categories from a set of training examples, each of them belonging to one of the categories. Relative to the other machine learning methods, SVMs are powerful methods at recognizing subtle patterns in complex datasets [23]. For diagnostic classification, an SVM classification scheme of CT/MRI texture analysis was used for differentiating glioma vs. primary central nervous system (CNS) lymphoma [16], renal cell carcinoma (RCC) vs. angiomyolipoma (AML) [24] and LN metastasis [25]. It has been reported that SVM based on MRI textural features was noninferior to expert human evaluation in the differentiation of CNS lymphoma and glioma [16]. You et al. reported SVM based on CT textural features (SVM on TF) of renal masses could accurately differentiate AML without visible fat from RCC [24]. Yang et al. reported that in predicting occult LN metastasis of lung cancer before surgery, the performance of the SVM on TF was higher than that of the model based on clinical-histopathologic features (i.e., age, sex, tumor location, diameter, and histology)[25]. Considering these classification abilities, we developed texture-based SVM classifier in differentiating focal-type AIP and PD in this study.

CT is one of the most commonly used imaging modalities for the diagnosis of pancreatic lesions. Although known to be difficult in the diagnosis of focal-type AIP based on imaging, previous studies reported that some imaging findings of CT were useful in distinguishing focal-type AIP from PD. These imaging findings contained; delayed homogeneous enhancement on dynamic CT, a hypoattenuating capsule-like rim, the presence of the “duct-penetrating” sign (mass penetrated by an unobstructed pancreatic duct), the absence of significant upstream main pancreatic duct (MPD) dilatation (> 5 mm), the absence of atrophic changes in the body and the tail of the pancreas, and enhanced duct sign (wall enhancement of MPD in the lesion) on multiphase contrast-enhanced CT [6, 26–29]. Furuhashi et al. reported that homogeneous enhancement during the portal phase, dotted enhancement during the pancreatic phase, duct-penetrating sign, enhanced duct sign and capsule-like rim were more frequently observed in focal-type AIP. On the other hand, ring-like enhancement during the delayed phase and peripancreatic strands with a length of at least 10 mm were more frequently observed in PD. Focal-type AIP was identified with 82% sensitivity and 98% specificity by using any four of these seven findings [26]. Although AIP was more likely to show a dotted enhancement in the pancreatic phase and a homogeneous enhancement in the portal phase, PD was more likely to show a heterogeneously decreased enhancement in the portal phase. In our study, we were able to objectively assess intralesional heterogeneity by using CT texture-based analysis to differentiate focal-type AIP and PD via a machine learning approach. Our results of CT texture analysis may support the findings of previous subjective studies.

In MRI, Choi et al. reported that the homogeneous enhancement (*p* = 0.001), duct penetrating sign (*p* < 0.001), and an ADC value less than 0.9407 × 10^–3^ mm^2^/s (*p* < 0.001) were significant for differentiating focal-type AIP from PD in multivariate analysis. When two of these three criteria were satisfied, 80% (12/15) of focal-type AIPs were identified with specificity of 98.7% [12]. 18F-FDG PET/CT findings have also been reported to differentiate AIP from PD (*p* < 0.05) with the AUCs of 0.700 (early SUV max) and 0.687 (delayed SUV max) in the previous study [14]. Although we developed SVM classifier based on only CT texture, a combination of the imaging features of these modalities may be more practical in clinical settings.

In our study, AUC, sensitivity (%), specificity (%), accuracy (%), PPV (%), and NPV (%) of our SVM classifier were 0.920, 100.0, 75.0, 90.0, 85.7, and 100.0%, respectively. Differentiation between focal AIP and PD should ideally be based on a combination of CT, MRI, and PET imaging findings, however, there are cases that are difficult to differentiate even with all these imaging findings. We believe that CT texture analysis can be a new quantitative tool in the differential diagnosis of pancreatic lesions as well as ADC values or SUV max. However, the performance of our learning model was lower than that of prior published work in differentiating AIP from PD with CT radiomics features [17]. In the previous study AIP patients included not only focal-type but also nearly diffuse type and diffuse type. Our study was limited to only focal type AIP and PD with surgical indication. This may be the cause of relatively low AUC values compared to previous studies.

**Fig. 6:**
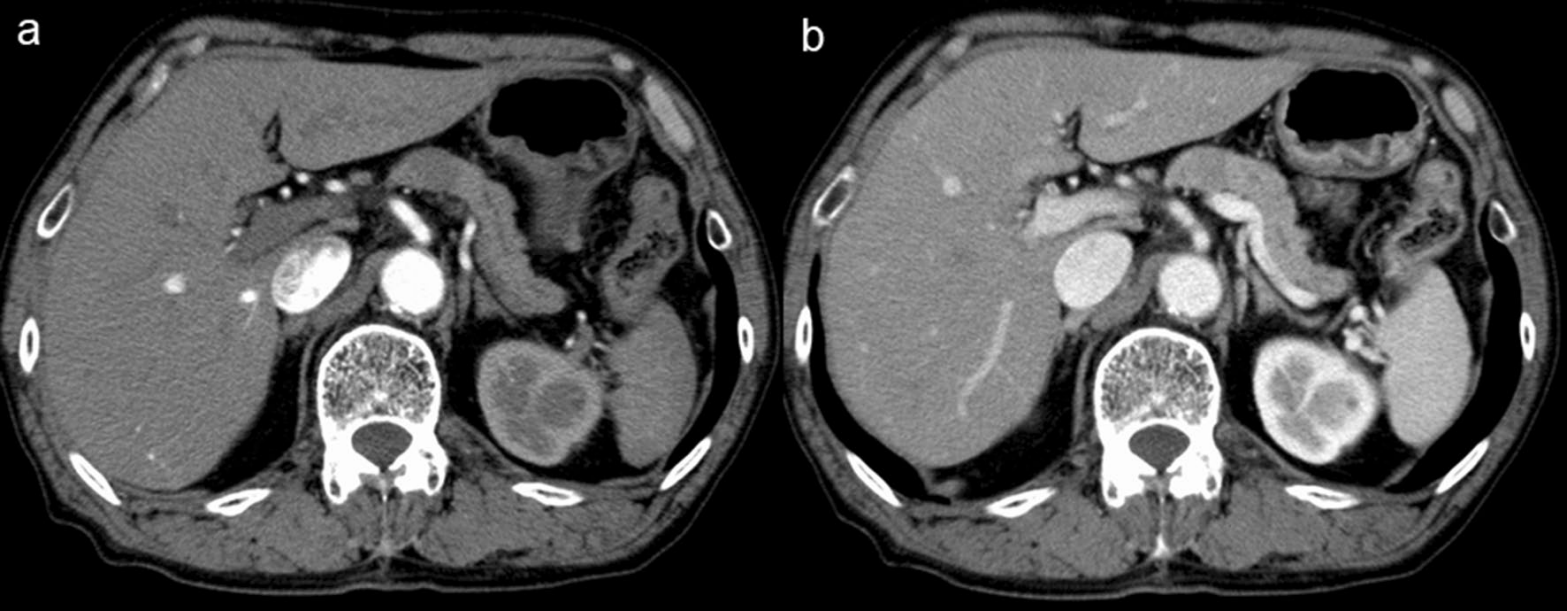
Arterial phase (**a**) and portal phase (**b**) images of the patient with focal-type autoimmune pancreatitis (AIP). The pancreatic lesion had an absence of atrophic changes in the body and the tail of the pancreas and an absence of significant upstream main pancreatic duct dilatation. The support vector machine (SVM) classified it as a pancreatic duct carcinoma (PD), while all radiologists classified the case correctly as AIP

Our SVM outputs could significantly improve reader’s performance including that of the expert readers. There were five cases in which all radiologists diagnosed correctly and the SVM failed to do so, and only one case in which all radiologists failed and the SVM diagnosed correctly. It is interesting to analyze the discrepancies between the SVM output and the radiologist’s interpretation, particularly for those cases in which the radiologists diagnosed correctly and the SVM failed to do so. Figure 6 shows the representative case of the SVM failure. In this case, the tumor had an absence of atrophic changes in the body and the tail of the pancreas and an absence of significant upstream MPD dilatation. This case was more typical of AIP, however, the SVM classified it as a PD. One cause of disagreement between radiologists and the SVM classifier may be that radiologists take into account the changes in the surroundings of lesions, such as atrophic changes in the body and the tail of the pancreas, upstream MPD dilatation, and a hypoattenuating capsule-like rim. Radiomics represents a method for the quantitative description of medical images. It is, however, important to note that radiomics should only be viewed as an additional tool and not as a standalone diagnostic algorithm [30]. Furthermore, we believe that ideally, machine learning should be used by radiologists applying it with other key image findings who have the knowledge of the limitation of radiomics.

Our study has several limitations. First, our study was a retrospective study with only a small number of patients. Second, the CT scanners used in our study were not uniform between patients and an inter-scanner difference may have affected the texture analysis results. Further studies with a larger study population and uniform CT scanner are needed to confirm the results of this study. Third, the readers reviewed only an axial image slice of the arterial and portal phase of the lesion in the observer performance study. This process might be influenced in readers performance. Fourth, in the present study, we constructed the SVM classifier to differentiate focal-type AIP and PD, though etiologies other than those diseases can also appear as pancreatic mass. Finally, lesion segmentation in our study was conducted manually by only one radiologist confirmed by the other radiologist because prior studies of CT texture analysis have shown good to excellent interobserver agreement [31–35]. However, a risk of subjective tendency or bias must be considered.

In conclusion, CT texture analysis via a machine learning approach can help clinicians differentiate focal-type AIP and PD. Where radiologists effectively incorporate machine learning methods into the clinical practices their diagnostic abilities will be extremely important.
