# Correction to: The effect of CT texture‑based analysis using machine learning approaches on radiologists’ performance in differentiating focal‑type autoimmune pancreatitis and pancreatic duct carcinoma

**DOI:** 10.1007/s11604-022-01313-x

**Published:** 2022-07-09

**Authors:** Kenta Anai, Yoshiko Hayashida, Issei Ueda, Eri Hozuki, Yuta Yoshimatsu, Jun Tsukamoto, Toshihiko Hamamura, Norihiro Onari, Takatoshi Aoki, Yukunori Korogi

**Affiliations:** 1 Department of Radiology, University of Occupational and Environmental Health, 1-1, Iseigaoka, Yahatanishi-ku, Kitakyushu, Fukuoka 807-8555 Japan; 2 Department of Radiology, Kyushu Rosai Hospital, Moji Medical Center, 3-1, Higashiminatomachi, Moji-ku, Kitakyushu, Fukuoka 801-8502 Japan

## Correction to: Japanese Journal of Radiology 10.1007/s11604-022-01298-7

In the original publication the name of the fifth author was incorrectly published as: “Yuuta Yoshimatsu”. The correct author name is Yuta Yoshimatsu as given in this correction.

Under Section: Texture feature extraction, the Reference number should read as [22] and hence, the sentence starting with: “We chose…” should read as:

We chose an axial image slice of arterial and portal phase on the basis of the maximum diameter of the lesion. Texture parameter calculation was performed with the software LIFEx ([22]; version 4.00, available at https://www.lifexsoft.org/).

In References, Reference [22] should read as:

22. Nioche C, Orlhac F, Boughdad S, Reuzé S, Goya-Outi J, Robert C, et al. LIFEx: a freeware for radiomic feature calculation in multimodality imaging to accelerate advances in the characterization of tumor heterogeneity. Cancer Res. 2018;78(16):4786–9.

